# An Allergic Lung Microenvironment Suppresses Carbon Nanotube-Induced Inflammasome Activation via STAT6-Dependent Inhibition of Caspase-1

**DOI:** 10.1371/journal.pone.0128888

**Published:** 2015-06-19

**Authors:** Kelly A. Shipkowski, Alexia J. Taylor, Elizabeth A. Thompson, Ellen E. Glista-Baker, Brian C. Sayers, Zachary J. Messenger, Rebecca N. Bauer, Ilona Jaspers, James C. Bonner

**Affiliations:** 1 Department of Biological Sciences, Environmental and Molecular Toxicology Program, North Carolina State University, Raleigh, North Carolina, United States of America; 2 Center for Environmental Medicine, Asthma, and Lung Biology, School of Medicine, University of North Carolina, Chapel Hill, North Carolina, United States of America; Virginia Tech University, UNITED STATES

## Abstract

**Background:**

Multi-walled carbon nanotubes (MWCNTs) represent a human health risk as mice exposed by inhalation display pulmonary fibrosis. Production of IL-1β via inflammasome activation is a mechanism of MWCNT-induced acute inflammation and has been implicated in chronic fibrogenesis. Mice sensitized to allergens have elevated T-helper 2 (Th2) cytokines, IL-4 and IL-13, and are susceptible to MWCNT-induced airway fibrosis. We postulated that Th2 cytokines would modulate MWCNT-induced inflammasome activation and IL-1β release *in vitro* and *in vivo* during allergic inflammation.

**Methods:**

THP-1 macrophages were primed with LPS, exposed to MWCNTs and/or IL-4 or IL-13 for 24 hours, and analyzed for indicators of inflammasome activation. C57BL6 mice were sensitized to house dust mite (HDM) allergen and MWCNTs were delivered to the lungs by oropharyngeal aspiration. Mice were euthanized 1 or 21 days post-MWCNT exposure and evaluated for lung inflammasome components and allergic inflammatory responses.

**Results:**

Priming of THP-1 macrophages with LPS increased pro-IL-1β and subsequent exposure to MWCNTs induced IL-1β secretion. IL-4 or IL-13 decreased MWCNT-induced IL-1β secretion by THP-1 cells and reduced pro-caspase-1 but not pro-IL-1β. Treatment of THP-1 cells with STAT6 inhibitors, either Leflunomide or JAK I inhibitor, blocked suppression of caspase activity by IL-4 and IL-13. *In vivo*, MWCNTs alone caused neutrophilic infiltration into the lungs of mice 1 day post-exposure and increased IL-1β in bronchoalveolar lavage fluid (BALF) and pro-caspase-1 immuno-staining in macrophages and airway epithelium. HDM sensitization alone caused eosinophilic inflammation with increased IL-13. MWCNT exposure after HDM sensitization increased total cell numbers in BALF, but decreased numbers of neutrophils and IL-1β in BALF as well as reduced pro-caspase-1 in lung tissue. Despite reduced IL-1β mice exposed to MWCNTs after HDM developed more severe airway fibrosis by 21 days and had increased pro-fibrogenic cytokine mRNAs.

**Conclusions:**

These data indicate that Th2 cytokines suppress MWCNT-induced inflammasome activation via STAT6-dependent down-regulation of pro-caspase-1 and suggest that suppression of inflammasome activation and IL-1β by an allergic lung microenvironment is a mechanism through which MWCNTs exacerbate allergen-induced airway fibrosis.

## Introduction

Carbon nanotubes (CNTs) are a product of the emerging nanotechnology industry and have numerous potential applications in structural engineering, electronics, and medicine [1,2]. Despite these benefits, CNTs represent an impending risk to human health as it has been shown that mice exposed to CNTs develop pulmonary inflammation and fibrosis following inhalation exposure [1–6]. Structurally, CNTs are graphene sheets rolled into cylinders that are one (“single-walled”, SWCNT) or multiple (“multi-walled”, MWCNT) layers thick. MWCNTs have unique physical and chemical properties that make them particularly hazardous, including a fiber-like shape with increased rigidity reinforced by multiple concentric layers and residual metal catalyst from the manufacturing process [1,2,7,8]. MWCNTs also have a high surface area per unit mass that allows for increased potential for ROS production and subsequent cellular damage [1,2,7,8].

There is particular cause for concern that engineered nanoparticles, including MWCNTs, could pose the greatest health risk to individuals with pre-existing lung disease, including asthma [1,3,5]. Asthma affects over 300 million individuals worldwide, and is characterized by periodic acute bronchospasms and chronic airway inflammation and remodeling [9]. Indoor allergens, in particular house dust mite (HDM), play an important role in the development of allergic asthma [10]. Allergic asthma has a specific inflammatory phenotype, characterized by eosinophils, CD4^+^ T cells, and T-helper 2 (Th2) cytokines such as IL-4 and IL-13 [10–12]. MWCNTs have been shown to exacerbate allergic airway inflammation, mucous cell metaplasia, and airway fibrosis in mice [5,13–15]. Moreover, transgenic mouse models indicate that specific genes (e.g., COX-2, T-bet) regulate susceptibility to MWCNT-induced exacerbation of allergic airway disease [16,17]. Collectively, these studies suggest that individuals with asthma would be more susceptible to the adverse respiratory effects of inhaled MWCNTs.

Macrophages play a key role in the lung by engulfing inhaled MWCNTs via phagocytosis and removing them from the lungs through the mucociliary escalator or lymphatic drainage [7]. Macrophage phenotype is modified by Th1 and Th2 immune microenvironments that induce classically-activated and alternatively-activated macrophage (CAM and AAM) phenotypes, respectively. Interferon-gamma (IFN-γ), increased in a Th1 microenvironment, induces a CAM phenotype, which is primarily involved in the innate pro-inflammatory immune response and microbial killing. Th2 cytokines, including IL-4 and IL-13, induce an AAM phenotype, which is involved in parasite killing, wound healing, allergy, susceptibility to pathogens, and the pathogenesis of fibrosis [18,19].

A variety of fiber-like particles, including asbestos, silica, and MWCNTs, all exert at least part of their pro-inflammatory activity by activating macrophage inflammasomes [20–23]. Fourteen different inflammasomes exist in humans, defined by the variant of NOD-like receptor (NLR) protein they encode [24]. The NLR pyrin domain containing 3 (NLRP3) inflammasome is focused around a specific NLR, NLRP3, and also contains two other subunits: an inactive caspase-1 (pro-caspase-1) and an ASC (PYCARD) adaptor [23,25]. Caspase-1 is a pro-inflammatory cysteine protease responsible for cleavage of immature inflammatory cytokines such as IL-1β and IL-18 into mature forms that are capable of being secreted. The ASC adaptor functions as a link between the NLRP3 protein and pro-caspase-1. Once NLRP3 is activated, oligimerization occurs, allowing NLRP3 to interact with ASC. The caspase-recruitment domain (CARD) of ASC can then interact with the CARD domain of pro-caspase-1, resulting in cleavage of pro-caspase-1 into active caspase-1, a p10/p20 tetramer. Once active, caspase-1 cleaves pro-IL-1β into a mature form that is subsequently secreted from the cell [25–27]. Functional caspase-1 is essential for cleavage of pro-IL-1β and subsequent secretion of mature IL-1β.

The impact of the asthma microenvironment on inflammasome activation by MWCNTs or any other agent has not been investigated. However, there is evidence that the Th2 cytokines that characterize the asthmatic microenvironment can modulate caspase-1. For example, Th2 cytokines, in particular IL-13, have been shown to inhibit caspase-1 activity. Niebuhr *et al* demonstrated that treatment of primary human monocytes with IL-13 suppressed caspase-1 activity, while Cihakova *et al* showed that IL-13^-/-^ mice have increased levels of caspase-1 activation [28,29]. The transcriptional profile of IL-13-treated human monocytes was also thoroughly examined by Scotton *et al*, who showed significant down-regulation of pro-caspase-1 mRNA following 8 hours of IL-13 exposure [30].

To our knowledge, there is currently no information on MWCNT-induced inflammasome activation in macrophages maintained in a Th2 microenvironment *in vitro* or in an experimental animal model of allergic asthma. However, a recent study showed that mRNA levels of inflammasome components and IL-1β are suppressed in sputum cells obtained from individuals with asthma or allergic rhinitis compared to normal individuals [31]. In the present study, we found that MWCNT-induced IL-1β secreted by a human monocytic cell line (THP-1 cells) *in vitro* or produced in the lungs of mice *in vivo* was suppressed by a Th2 microenvironment, and this corresponded with decreased pro-caspase-1 *in vitro* and *in vivo*. Despite reduced IL-1β secretion, mice exposed to HDM and MWCNTs had more severe airway fibrosis than mice exposed to HDM or MWCNTs alone, suggesting that suppressing inflammasome activation could result in a greater fibrogenic response to nanomaterials.

## Materials and Methods

### Carbon nanotubes

Multi-walled carbon nanotubes (MWCNTs) were purchased from Cheap Tubes, Inc. (Brattleboro, VT). We previously performed thorough size and zeta potential characterization of these MWCNTs through the National Institute of Environmental Health Science (NIEHS) NanoGo Consortium [32]. Characterization was performed on samples of dry MWCNTs, as well as MWCNTs suspended in water or the tissue culture medium used to culture THP-1 cells (RPMI). The primary size dimensions of the MWCNTs measured by TEM were 20–30 nm diameter and 5–10 μm length. Aggregate size of MWCNTs measured by dynamic light scattering (DLS) was 324±33 nm in water and 419±48 nm in RPMI. Zeta potential measured by zetasizer was -12.1±0.3 mV in water and -10.5±0.9 mV in RPMI. MWCNTs in powder form were weighed out using a milligram scale (Mettler, Toledo, OH) and re-suspended in a sterile, 0.1% pluronic F-68 (Sigma-Aldrich, St. Louis, MO) in Dulbecco’s phosphate buffered saline (DPBS) solution to obtain a final concentration of 5 mg/mL. The suspended nanoparticles were then dispersed using a cuphorn sonicator (Qsonica, Newton, CT) for one minute at room temperature prior to cell dosing or delivery to the lungs of mice by oropharyngeal aspiration as described below. For experiments with THP-1 cells *in vitro*, the MWCNTs were further diluted in RPMI. The endotoxin content of MWCNT stock suspensions was previously tested using the colorimetric Limulus amebocyte lysate assay (Lonza Inc., Allendale, NJ). The LPS content of all ENM suspensions was < 0.3 EU/ml [32].

### Cell culture

Human monocyte cells (THP-1) were purchased from ATCC (Manassas, VA) (Cat#: TIB-202). Cells were cultured in suspension in RPMI-1640 Medium (Invitrogen, Carlsbad, CA) supplemented with 10% fetal bovine serum (FBS) (Gibco, Carlsbad, CA) at 37°C and 5% CO_2_. The THP-1s were grown to confluence and differentiated into macrophage-like cells using 150 nM of 1α,25-Dihydroxy-Vitamin D_3_ (EMD Millipore, Billerica, MA) for 24 hours. Once the cells were semi-adherent, 160 nM of phorbol 12-myristate 13-acatate (PMA) (LC Laboratories, Woburn, MA) in sterile dimethyl sulfoxide (DMSO) (Sigma-Aldrich) was applied to the cells for 30 minutes to initiate maturation from monocyte to macrophage [33]. Following cell maturation, 2 μg/mL lipopolysaccharide (LPS) (Sigma-Aldrich) was added to the cells, following by 10 ng/mL recombinant human IL-13 (#213-IL/CF) or IL-4 (#204-IL/CF) (R&D Systems, Inc., Minneapolis, MN) and/or MWCNTs. Once dosed, cell mixtures were aliquoted into 96 or 48-well plates (Thermo Fisher Scientific, Waltham, MA). Cell supernatants, RNA, and whole cell protein were collected 24 hours post-MWCNT exposure.

### Transmission electron microscopy

THP-1 cells exposed to MWCNTs (10 μg/mL) were post-fixed in 1% osmium tetroxide in 0.1M sodium phosphate buffer, pH 7.2, dehydrated through graded ethanol solutions, cleared in acetone, and then infiltrated and embedded in Spurr’s resin. Unstained thin sections were then mounted on copper grids and examined using a Philips EM208S transmission electron microscope.

### Animals and experimental design

Pathogen-free, 6 to 8 week-old, male wild type (WT) C57BL/6 mice were obtained from The Jackson Laboratory (Bar Harbor, ME). Mice were sensitized intranasally with house dust mite (HDM) allergen (0.5 μg/mL) (Greer Laboratories, Lenoir, NC) or sterile saline under an isoflurane anesthetic five days a week for two weeks. Following allergen sensitization, mice were exposed to MWCNTs (2 mg/kg) or sterile saline via oropharyngeal aspiration under an isoflurane anesthetic. Mice were euthanized 1 day or 21 days after MWCNT exposure via intraperitoneal injection of 0.3 mL pentobarbital (Fatal Plus) (Vortech Pharmaceuticals, Dearborn, MI).

### Tissue collection

Lungs were serially lavaged two times with 0.5 mL of DPBS and combined. An aliquot from each sample was immediately used to analyze differential cell counts while the remaining sample was stored at −80°C. A Thermo Scientific Cytospin 4 Cytocentrifuge (Thermo Fisher Scientific) was used to spin cells from the BALF of each animal onto glass slides. Samples were then fixed and stained with the Diff-Quik Stain Set (Dade Behring, Inc., Newark, DE). Differential cell counts were performed on at least 500 cells per sample and represented as the mean ± SEM per exposure group. Total inflammatory cell counts were also performed, counting three frames per slide at 10x magnification. After collection of BALF, the middle and caudal lobes of the right lung, as well as the heart, spleen, and a section of the liver, were stored in RNAlater according to the manufacturer’s instructions (Ambion, Austin, TX), and used for quantitative RT-PCR analysis. The cranial lobe of the right lung was flash frozen in liquid nitrogen and stored at −80°C for protein evaluation. The left lung was infused with 10% neutral buffered formalin, fixed for 24 hours, transferred to 70% ethanol, and embedded in paraffin. Three cross-sectional sections of tissue were cut and processed for histopathology with a hematoxylin and eosin (H&E), Alcian blue/periodic acid-Schiff (AB/PAS), and/or Masson’s trichrome stain. Whole blood was collected from the jugular vein and allowed to coagulate for approximately 15 minutes in Serum Separator Tubes (BD Microtainer, Franklin Lakes, NJ) prior to centrifugation for serum collection. Serum was then stored at -80°C.

### Semi-quantitative morphometric analysis

#### Lung Inflammation

Three sections of formalin-fixed lung tissue from each 1 day animal were analyzed under light microscopy and scored for inflammation in a blinded fashion. Lungs were scored on a scale of 1–5 based on inflammatory cell infiltration, alveolar wall thickening, and the manifestation of extracellular matrix. Animals were scored with 1 representing the saline control animals, 2 representing very minimal inflammation, 3 representing mild inflammation, 4 representing moderate inflammation, and 5 representing very severe inflammation [16]. Data was shown as the mean value ± SEM for 11–14 animals per treatment group.

#### Mucous Cell Metaplasia

Mucin production and mucous cell metaplasia were analyzed utilizing a previously published protocol [34]. Images of Alcian blue/PAS stained lung sections were captured and ImageJ software version 1.44o (National Institutes of Health) was utilized to quantify mucin levels in the airways. Positively stained cells were analyzed by a de-convolution module using a threshold method and then standardized for measurement in microns.

#### Airway Fibrosis

Quantification of the thickness of collagen surrounding airways was performed according to a previously published airway intersect method [17]. Briefly, photomicrographs of trichrome-stained sections of lung tissue containing circular to oval-shaped small or medium-sized airways were captured using a 10X objective on an Olympus BX41 microscope (Olympus America Inc., Center Valley, PA) and digitized. The thickness of the collagen layer surrounding the airways was measured using the ruler tool in Adobe Photoshop CS3 extended program (Adobe Systems, Inc., San Jose, CA) at eight equidistant points and averaged. To validate the airway intersect measurements, a second independent method similar to a previously published method [34] was used to measure the airway collagen area corrected for length of basement membrane (i.e., area/perimeter ratio). Briefly, the lasso tool in Adobe Photoshop was used to surround the trichrome-positive collagen around an airway (outer area). A second measurement was made by surrounding the basement membrane of the same airway (inner area) and the length of the airway circumference (i.e., perimeter) was also derived from this measurement. The difference between the outer and inner area was defined as the ‘area’ and divided by the ‘perimeter’ to derive area/perimeter measurements. Both methods were performed in a blinded manner, where the treatment group was unknown to the observer scoring the sections. Five airways per animal were analyzed in a random, blinded manner, and the data were expressed as the mean ± SEM of five animals per treatment group per time point.

### ELISA

Quantikine and DuoSet ELISA kits (R&D Systems, Inc.) were utilized to measure IL-1β levels in THP-1 cell culture medium (#DLB50) and mouse BALF (#DY401). Cell culture conditioned medium collected 24 hours post-MWCNT and IL-13 or IL-4 exposure was used for the assay. Cell culture samples were diluted 100-fold in DPBS for IL-1β analysis and assayed following the manufacturer’s protocol. Mouse BALF was left undiluted and assayed for IL-1β following the manufacturer’s protocol. Mouse serum was diluted 1:10 in sterile DPBS and IgE was analyzed using a mouse IgE ELISA kit (#557079) (BD Pharmingen, San Jose, CA). Absorbance was measured at 450nm by the Multiskan EX microplate spectrophotometer (ThermoFisher Scientific) with a correction wavelength of 540nm. IL-1β and IgE concentrations were interpolated from a standard curve using linear regression analysis. Values were expressed as mean±SEM.

### Quantitative real-time RT-PCR

Taqman Quantitative RT-PCR (qRT-PCR) was utilized to measure mRNA levels in THP-1 monocytes and whole mouse lungs. Cell culture RNA was collected 24 hours post-MWCNT exposure using the Zymo Research Quick-RNA MiniPrep Kit (Genesee Scientific, San Diego, CA) and following the manufacturer’s protocol. 20 μL or 40 μL lysis buffer was added to each well in the 96-well or 48-well plate, respectively, and columns of wells were scraped and combined to form one sample. Right cranial and caudal lobes from each mouse lung were homogenized and whole lung RNA extracted and purified using the Zymo Research Quick-RNA MiniPrep Kit as well. RNA concentrations for each sample were quantified using a Nanodrop 2000 Spectrophotometer (ThermoFisher Scientific) and each sample was normalized to a final concentration of 25ng/μL in RNAse-free H_2_O. qRT-PCR was performed utilizing reagents from the SuperScript III Platinum One-Step qRT-PCR Kit (Life Technologies, Grand Island, NY) on a StepOne Plus instrument (Applied Biosystems, Foster City, CA). A comparative C_T_ technique was used to quantify target gene expression. Primers for human pro-IL-1β (Hs01555410_m1) and pro-caspase-1 (Hs00354836_m1) were utilized to analyze THP-1 mRNA levels. Primers for mouse pro-IL-1β (Mm01336189_m1), IL-13 (Mm00434204_m1), IL-4 (Mm00445259_m1), IL-5 (Mm00439646_m1), CXCL1 (Mm04207460_m1), CXCL2 (Mm00436450_m1), CCL2 (Mm00441242_m1), PDGF-A (Mm00833533_m1), PDGF-B (Mm01298578_m1), and TGF-β1 (Mm03024053_m1) were utilized to analyze whole mouse lung mRNA. THP-1 and mouse lung mRNA expression were normalized against the endogenous control β-2-microglobulin for either human (Hs00984230_m1) or mouse (Mm00437762_m1), respectively, and measured relative to vehicle-treated controls for both cell culture and mouse samples. All qRT-PCR primers were purchased from Life Technologies. Each sample was analyzed in duplicate and the StepOne Plus software used to calculate relative quantitation values and express them as fold-change over controls. Fold change was expressed as mean ± SEM.

### Western blotting

Whole cell lysates and whole lung protein were isolated from THP-1 cells and snap-frozen mouse lungs, respectively. Protein concentrations were analyzed using the Pierce BCA Protein Assay Kit (ThermoFisher Scientific). Absorbance was measured at 560nm and protein concentrations were interpolated from a standard curve using linear regression analysis and values were expressed as mean ± SEM. Each sample was then normalized to a final concentration of 10 μg protein in 15 μl lysis buffer. Samples were then separated on 4–12% SDS-PAGE gels (Life Technologies) and transferred to PVDF membranes. Membranes were blocked in 5% nonfat milk in TBS-T (20 mM Tris, 137 mM NaCl, and 0.1% Tween-20) and incubated in primary antibody (1:1000 dilution) overnight at 4°C. Primary antibody incubation was followed by incubation in horseradish peroxidase-conjugated secondary antibody (1:2500 dilution). Immunoblot signals were identified using enhanced chemiluminescence (ECL) (ThermoFisher Scientific). Densitometry was performed to quantify Western blotting signals as previously described [17]. Mouse monoclonal pro-caspase-1 (sc-56036) and rabbit polyclonal pro-IL-1β (sc-7884) primary antibodies were purchased from Santa Cruz Biotechnology, Inc. (Dallas, TX). Polyclonal rabbit total STAT-6 (#9362), polyclonal rabbit phosphorylated STAT-6 (#9361), and polyclonal rabbit β-actin (#4967) primary antibodies were purchased from Cell Signaling Technology (Beverly, MA). Anti-rabbit (#7074) and anti-mouse (#7076) secondary antibodies were purchased from Cell Signaling.

### Caspase-1 activity assay

THP-1 cells were grown to confluence and differentiated into macrophage-like cells using 150 nM of 1α,25-Dihydroxy-Vitamin D_3_ for 24 hours. Once the cells were semi-adherent, 160 nM of phorbol 12-myristate 13-acatate (PMA) in sterile DMSO was applied to the cells for 30 minutes to initiate maturation from monocyte to macrophage. Following maturation, THP-1 cells were treated for one hour with 50 μM Leflunomide in DMSO, a selective STAT6 inhibitor (Enzo Life Sciences, Farmingdale, NY) [35] or 4 μM InSolution JAK Inhibitor I in DMSO, a broad JAK/STAT inhibitor (EMD Millipore) [36]. THP-1 cells were then primed with LPS and exposed to MWCNTs and/or the combination of recombinant IL-4 and IL-13 for 24 hours. Cell supernatants were removed and cells washed with sterile DPBS. After washing, THP-1 cells were exposed to 50mJ UVB to induce apoptosis. Following UVB treatment, fresh media was added and cells were allowed to incubate at 37°C for 16 hours. Apoptotic cells were then collected and lysed, and analysis of caspase-1 activity was performed using the Caspase-1 Colorimetric Assay Kit from R&D Systems (#BF14100) and following the manufacturer’s protocol.

### Pro-caspase-1 immunohistochemical staining

Lung sections were fixed in 10% neutral buffered formalin for 24 hours followed by 70% ethanol before being embedded in paraffin. Tissue sections (5 μM) were deparaffinized, hydrated, treated with 3% H_2_O_2_, and subjected to antigen retrieval with a citrate buffer (pH 6.0) using a 2100-Retriever purchased from Electron Microscopy Sciences (Hatfield, PA). Sections were blocked with 10% normal horse serum for 1 hour at room temperature before being incubated with mouse monoclonal anti-pro-caspase-1 antibody (sc-56036; 1:2000) (Santa Cruz Biotechnology, Inc.) at 4°C for 20 hours. Sections were then incubated with biotinylated anti-mouse IgG for 30 minutes at room temperature and staining was detected using the mouse Vectastain Elite ABC kit (PK-6102) (Vector Laboratories, Burlingame, CA) and 3,3’-diaminobenzidine (DAB) (BioGenex, Fremont, CA) following the manufacturer’s protocol. The sections were counterstained with Mayer’s Hematoxylin (Sigma-Aldrich), dehydrated, and mounted. No pro-caspase-1 staining was observed when the primary antibody was omitted and the control normal horse serum was applied. CASP-1^-/-^ mice, purchased from Jackson Laboratories and utilized by Bauer, *et al*, acted as a negative control [37]. Pro-caspase-1 staining was quantified utilizing ImageJ software. Images of stained airways were captured at 10X magnification and positively stained cells and epithelium were analyzed by a de-convolution module using a threshold method and then standardized for measurement [34]. Final results were calculated as percent area of stained tissue relative to the total area of the image used. Three airways were quantified per lung section for statistical analysis.

### Statistical analysis

All data was collected and transformed into graphs, and statistical analysis was performed using GraphPad Prism software version 5.00 (GraphPad Software Inc., San Diego, CA). One-way ANOVA with a *post hoc* Tukey or unpaired Student’s t-test were used to determine significant differences between controls and treatments, and two-way ANOVA with a post-Bonferroni test was used to determine significant differences between treatment groups. Significance was set at *p* < 0.05 unless otherwise indicated. All 1 day animal data is representative of three replicate experiments, while all 21 day animal data is representative of two replicate experiments.

### Ethics statement

Mice were housed in a temperature and humidity controlled facility and given food and water *ad libitum*. All procedures involving animal use were approved by the Institutional Animal Care and Use Committee (IACUC) at North Carolina State University (protocol no. 13-086-B). No unfavorable health or behavior effects were observed during the animal studies.

## Results

### MWCNT-induced IL-1β secretion by LPS-primed THP-1 cells is inhibited by Th2 cytokines *in vitro*

**Fig 1:**
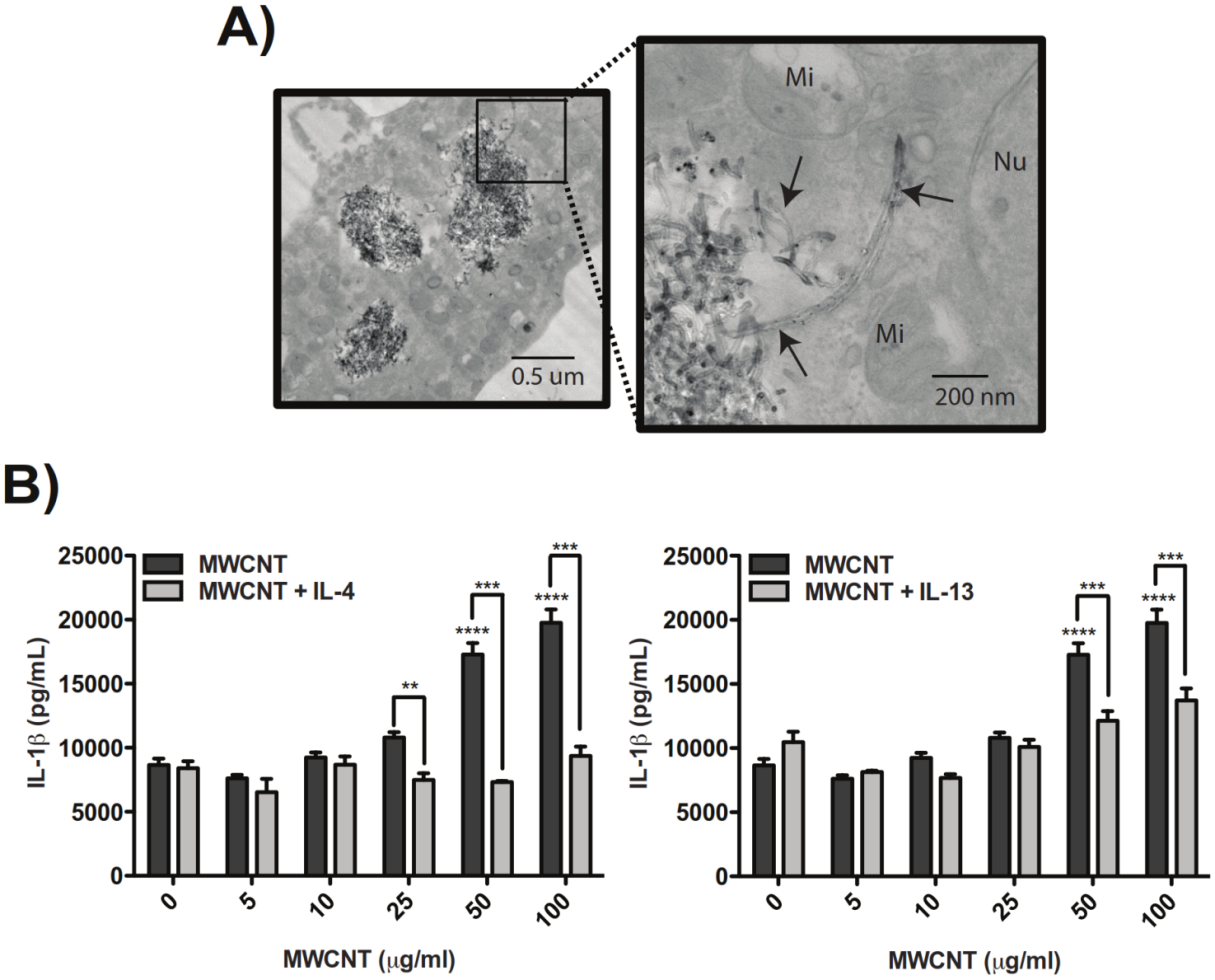
MWCNT-induced IL-1β secretion by LPS-primed THP-1 cells is inhibited by Th2 cytokines *in vitro* and corresponds to alternative macrophage activation. (A) Transmission electron micrographs demonstrating phagocytosis of MWCNTs by THP-1 cells after exposure to 10 μg/mL MWCNT for 24 hours (Mi = mitochondria, Nu = nucleus). Images taken at 14000X and 56000X, respectively. (B) Secreted IL-1β measured by ELISA in supernatants from LPS-primed THP-1 cells after exposure to MWCNTs for 24 hours and suppression of MWCNT-induced IL-1β secretion by co-incubation with 10 ng/ml IL-13 or IL-4. Data for MWCNTs alone is the same for both graphs. Statistical analysis was performed using a one-way ANOVA with a *post hoc* Tukey. ****P < 0.0001 for MWCNT treatment compared to untreated control. ***P < 0.001, **P < 0.01 for IL-13 or IL-4 co-exposure compared to MWCNTs alone. Graphs are representative of multiple experiments.

THP-1 monocytes were differentiated to macrophages with Vitamin D3 and PMA, primed with LPS, and exposed to MWCNTs in the absence or presence of IL-4 or IL-13 for 24 hours prior to collection of supernatants for measurement of IL-1β by ELISA. Transmission electron microscopy showed that THP-1 cells exhibited a macrophage-like morphology and avidly engulfed MWCNTs (Fig 1A). Aggregates of MWCNTs and singlet MWCNTs in cells visualized by TEM were consistent with the sizes reported in Methods and described previously [32]. Treatment of differentiated, LPS-primed THP-1 cells with MWCNTs caused a dose-dependent increase in secreted IL-1β in cell supernatants, and co-treatment with IL-4 or IL-13 (10 ng/ml) resulted in significant suppression of MWCNT-induced IL-1β secretion (Fig 1B). IL-4 was more potent than IL-13 in suppressing MWCNT-induced IL-1β secretion. While MWCNTs were observed in THP-1 cells pre-treated with IL-4 or IL-13, we cannot completely rule out that differences in uptake of MWCNTs could partially account for reduced inflammasome activation in cells pre-treated with these Th2 cytokines.

### Th2 cytokines suppress pro-caspase-1 without affecting levels of pro-IL-1β

**Fig 2:**
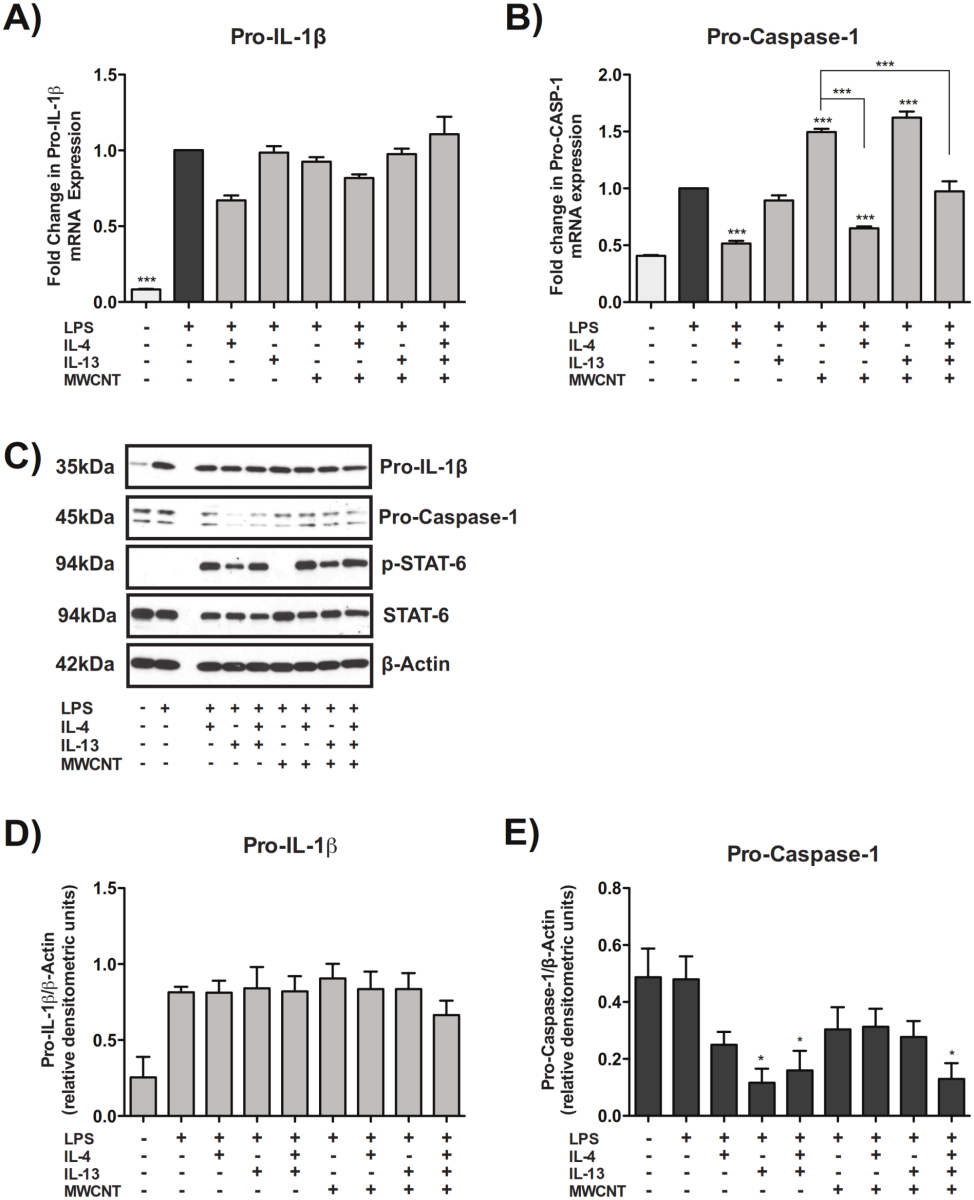
Th2 cytokines suppress pro-caspase-1 without affecting levels of pro-IL-1β. (A) Taqman quantitative RT-PCR results showing mRNA expression of pro-IL-1β in THP-1s exposed to 100 μg/mL MWCNTs with IL-4, IL-13, or IL-4 and IL-13 co-treatment. B) Taqman qRT-PCR results showing mRNA levels of pro-caspase-1 in THP-1s exposed to MWCNTs with IL-4, IL-13, or IL-4 and IL-13 co-treatment. Statistical analysis was performed using a one-way ANOVA with a *post hoc* Tukey. ***P < 0.001 for comparison between treatment groups. Values directly above bars indicate significant difference from LPS treatment alone. Values above connecting lines indicate significant difference compared to MWCNT treatment. Data is pooled from three replicate experiments. (C) Representative Western blots showing protein levels of pro-IL-1β, pro-caspase-1, phosphorylated (p)-STAT-6, STAT-6, and β-actin after 24 hour exposure of LPS-primed THP-1 cells to MWCNTs in the absence or presence of IL-4, IL-13, or a combination of IL-4 and IL-13. (D) Quantitative densitometric analysis of pro-IL-1β Western blot signal from two replicate experiments. (E) Quantitative densitometric analysis of pro-caspase-1 Western blot signal from three replicate experiments. Statistical analysis performed using an unpaired student t-test. *P < 0.05 compared to LPS priming alone.

LPS priming strongly induced levels of pro-IL-1β mRNA and protein as measured by Taqman qRT-PCR and by Western blotting, respectively (Fig 2A, 2C, and 2D). MWCNTs or Th2 cytokines did not change levels of LPS-induced pro-IL-1β mRNA or protein. Priming of THP-1 cells with LPS increased mRNA levels of pro-caspase-1 approximately two-fold and treatment of LPS-primed THP-1 cells with MWCNTs further increased mRNA levels of pro-caspase-1 (Fig 2B). Co-treatment with IL-4 or IL-4 and IL-13, but not IL-13 alone, reduced MWCNT-induced pro-caspase-1 mRNA levels. Treatment of THP-1 cells with MWCNTs and/or IL-13 or IL-4 did not alter mRNA levels of NLRP3 or PYCARD, two other key components of the NLRP3 inflammasome (data not shown). LPS priming alone did not increase protein levels of pro-caspase-1 as determined by Western blotting (Fig 2C and 2E). Treatment with MWCNTs alone increased levels of STAT6, but not phospho-STAT6, as exposure to IL-4 and/or IL-13 was necessary for phosphorylation of STAT6 (Fig 2C). However, both IL-4 and IL-13 suppressed protein levels of pro-caspase-1 as determined by densitometric evaluation of Western blots (N = 3) for pro-caspase-1 that were normalized against β-actin (Fig 2E).

### Inhibition of STAT6 in THP-1 cells increases caspase-1 activity

**Fig 3:**
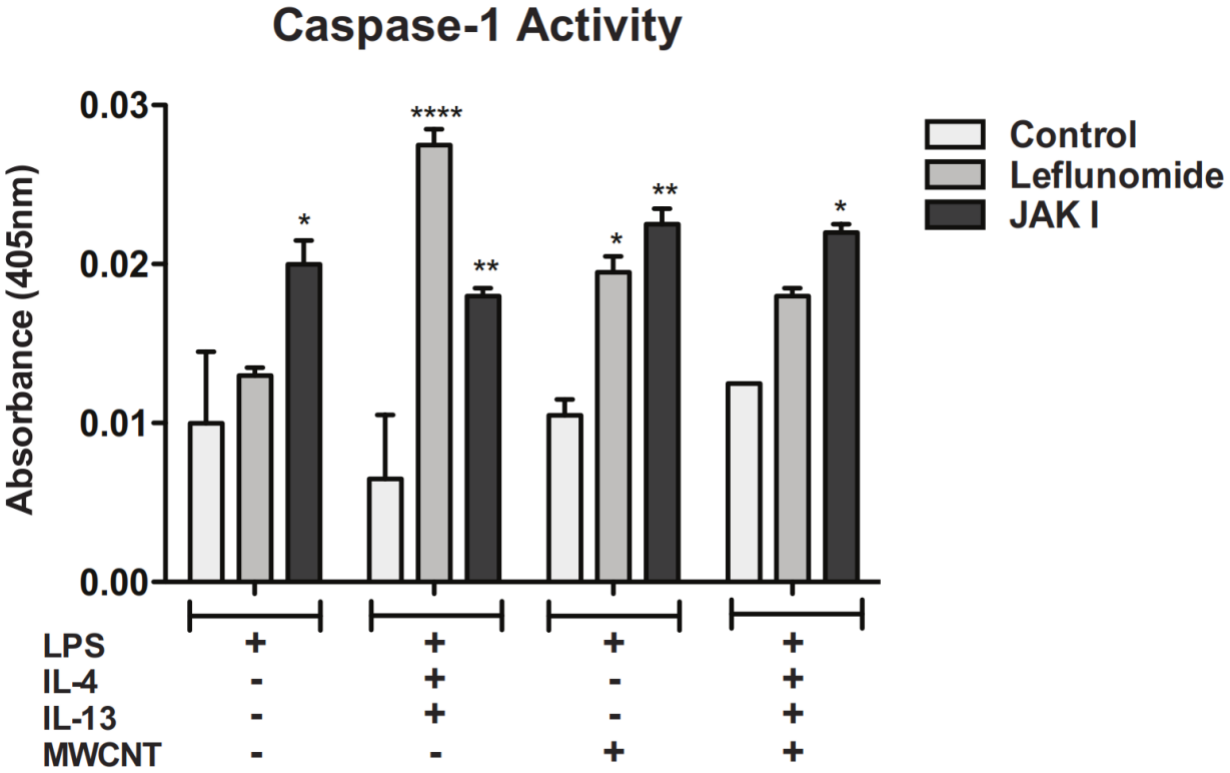
Inhibition of STAT6 in THP-1 cells increases activity of cleaved, active caspase-1. Caspase-1 activity assay measuring cleaved, active caspase-1 in cell lysates from THP-1 cells pre-treated with Leflunomide or JAK Inhibitor I, primed with LPS, and exposed to IL-4 and IL-13 and/or MWCNTs. Statistical analysis was performed using a one-way ANOVA with a *post hoc* Tukey. *P < 0.05, **P < 0.01, ****P < 0.0001 for inhibitor-treated cells compared to control within each treatment group. Data is representative of duplicate experiments.

Treatment of LPS-primed THP-1 cells with either Leflunomide, a STAT6–specific inhibitor, or JAK Inhibitor I (JAKI), a broad JAK/STAT inhibitor, blocked the suppression of caspase-1 activity by IL-4/IL-13 treatment (Fig 3). Moreover, Leflunomide or JAKI increased levels of caspase-1 activity significantly above controls.

### MWCNTs enhance the lung inflammatory response in mice sensitized to house dust mite allergen but reduce relative numbers of neutrophils

**Fig 4:**
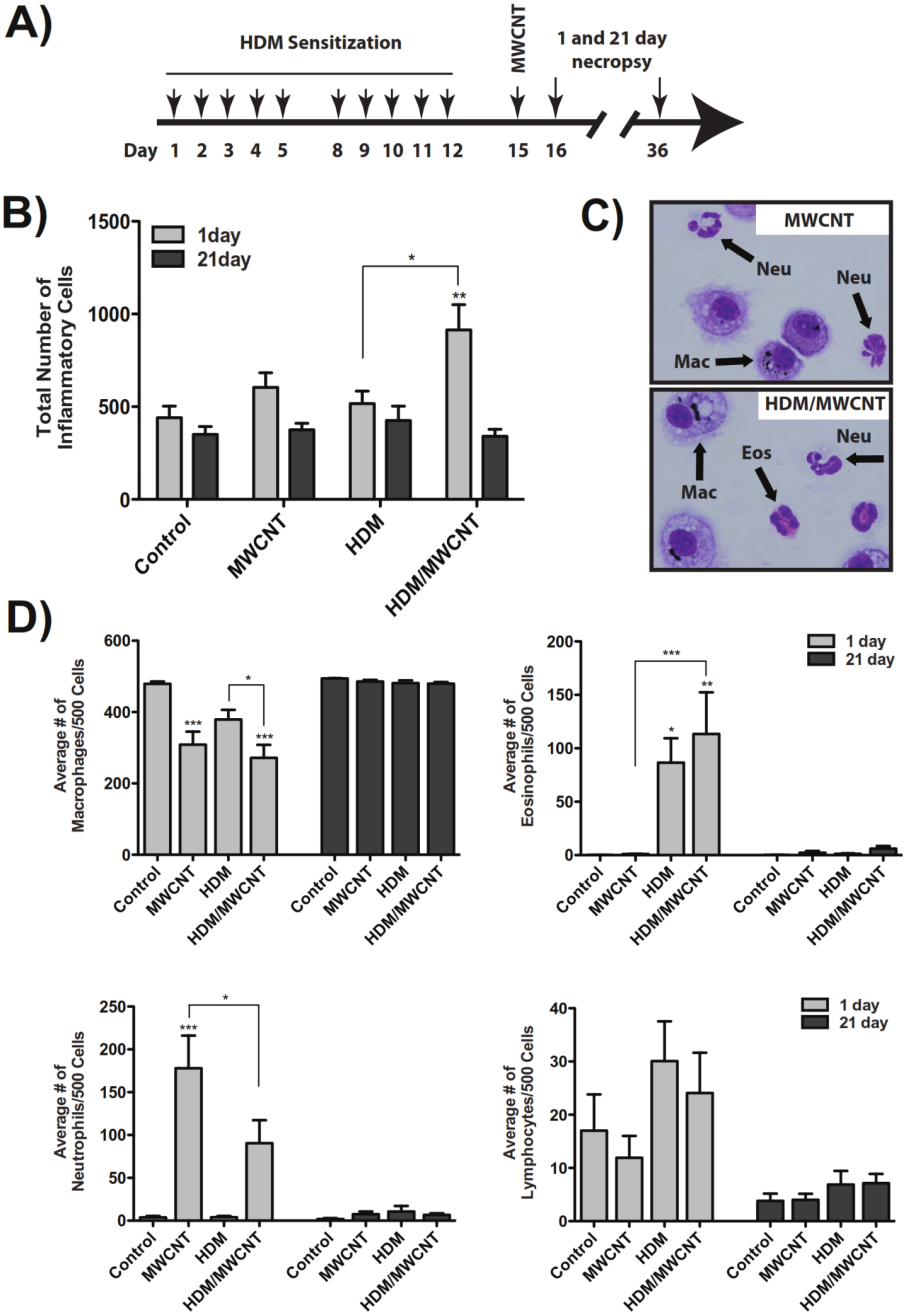
Effect of MWCNTs on the lung inflammatory response in mice after sensitization with HDM allergen. (A) Experimental protocol. (B) Total inflammatory cell numbers in the BALF at 1 day and 21 days in all treatment groups. ****P < 0.0001 for HDM/MWCNT mice compared to control animals. **P < 0.01 for HDM/MWCNT mice at 1 day compared to 21 days. (C) Cytospins of BALF showing neutrophilic inflammation with MWCNTs alone and mixed neutrophilic/eosinophilic inflammation with MWCNTs after HDM sensitization. Images taken at 100X magnification. (D) Relative number of inflammatory cell types counted in BALF cytospins at 1 and 21 days after MWCNT exposure with or without HDM. ***P < 0.001, **P < 0.01, *P < 0.05. Values directly above bars indicate significant differences compared to control animals. Values above connecting lines indicate significant difference between MWCNT, HDM, or HDM/MWCNT treatment groups. Statistical analysis for total cell counts and cell differentials performed using a one-way ANOVA with a *post-hoc* Tukey. N = 11–14 animals for all 1 day cell differentials and counts. N = 8–13 animals for all 21 day cell differentials and counts.

As illustrated in Fig 4A, C57BL6 mice were sensitized to HDM allergen and exposed to 2 mg/kg MWCNTs via oropharyngeal aspiration (OPA), then euthanized at 1 day or 21 days post-MWCNT exposure for collection of BALF and lung tissue. Mice exposed to the combination of HDM and MWCNTs had significantly increased total cell counts in BALF at 1 day post-MWCNT exposure compared to mice that received vehicle control or mice that received HDM alone (Fig 4B). The increase in the total number of inflammatory cells returned to control levels by 21 days post-MWCNT exposure. Cytospins of BALF from mice treated with HDM and MWCNTs showed a mixed neutrophilic/eosinophilic inflammatory response at 1 day compared to the eosinophilic response observed in mice sensitized to HDM alone or the neutrophilic response seen in mice exposed to MWCNTs alone (Fig 4C and 4D). BALF cell differential counts at 1 day showed a relative decrease in macrophage counts compared to an increase in the percentage of neutrophils in mice exposed to MWCNTs and an increase in the percentage of eosinophils in mice sensitized to HDM (Fig 4D). Interestingly, the relative percentage of neutrophils induced by MWCNTs was reduced by approximately half in mice pre-sensitized with HDM, while the relative percentages of eosinophils was approximately the same in mice treated with HDM or HDM and MWCNTs. Lymphocyte counts were not notably different between any treatment groups (Fig 4D). These inflammatory cell changes in BALF returned to nearly control levels by 21 days post-MWCNT exposure.

### Sensitization to HDM allergen followed by MWCNT exposure enhances lung inflammation and increases IL-1β lung mRNA, but suppresses MWCNT-induced IL-1β secretion in the lungs of mice

**Fig 5:**
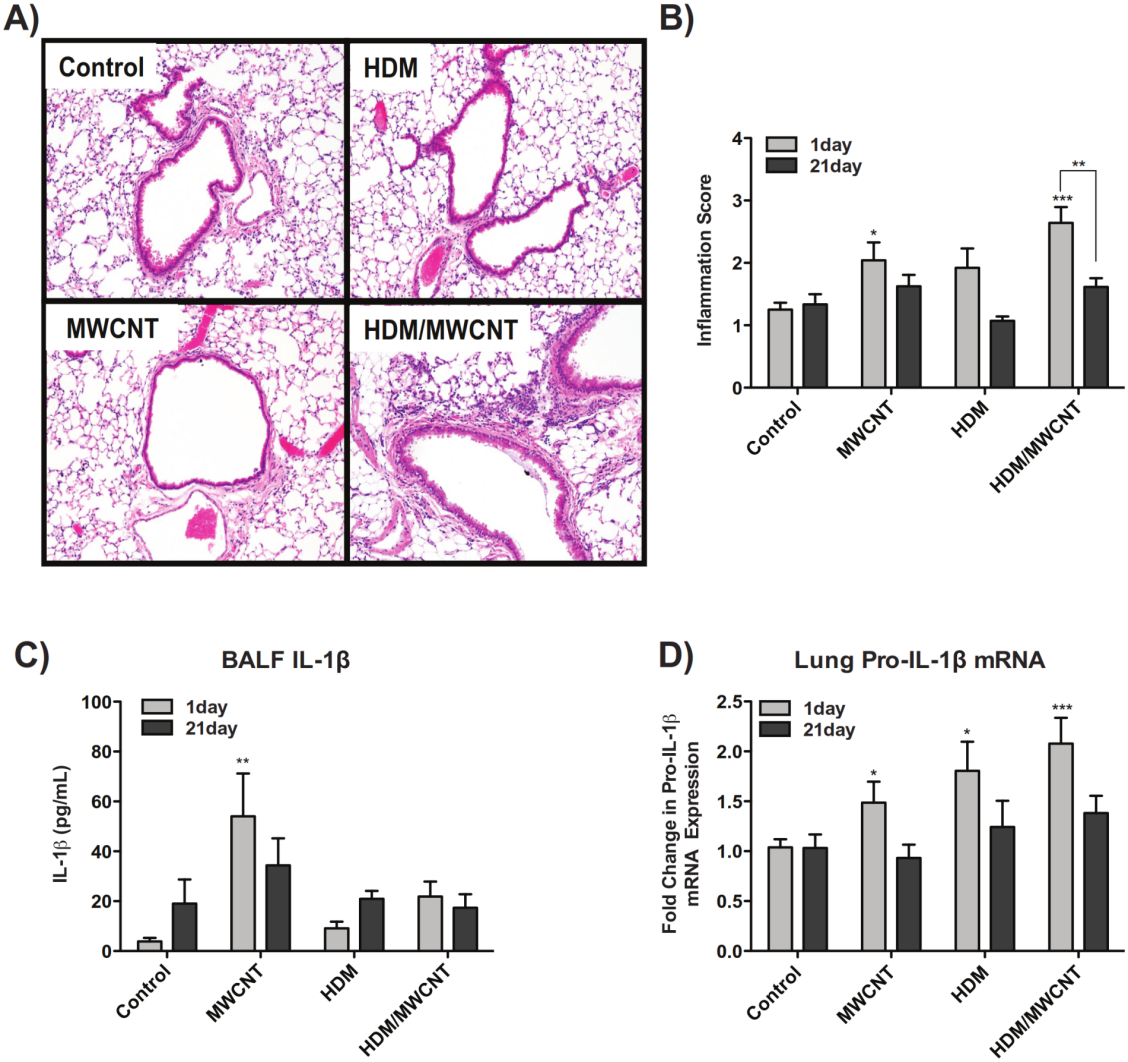
Effect of HDM sensitization and MWCNT exposure on allergic inflammation and inflammasome activation. (A) Representative hematoxylin and eosin (H&E)-stained lung sections from mice sensitized with HDM and exposed to MWCNTs for 1 day. Images taken at 10X magnification. (B) Qualitative inflammation scoring for mice sensitized to HDM and exposed to MWCNTs at 1 and 21 days. Lung sections from each treatment group scored in a blinded fashion according to severity of inflammation. Statistical analysis performed using a one-way ANOVA with a *post-hoc* Tukey for comparison within timepoints, and using a two-way ANOVA with Bonferroni post-tests for comparison between timepoints. ***P < 0.001, *P < 0.05 for treatment groups compared to control animals. **P < 0.01 for comparison between timepoints. (C) Levels of secreted IL-1β in the lung BALF of mice at 1 day and 21 days post-exposure to MWCNTs with or without pre-sensitization with HDM allergen. Statistical analysis performed using a one-way ANOVA with a *post-hoc* Tukey. **P < 0.01 compared to control animals. (D) Taqman qRT-PCR results showing lung mRNA levels of pro-IL-1β. Statistical analysis performed using an unpaired student t-test. ***P < 0.001, *P < 0.05 in treatment groups compared to control mice. N = 11–14 animals for all 1 day results, and N = 8–13 animals for all 21 day results.

Histopathological analysis of lung inflammation supported the results seen with the inflammatory cell counts, as hematoxylin and eosin (H&E)-stained lung sections from mice exposed to the combination of HDM and MWCNTs at 1 day had decidedly more inflammation than mice exposed to HDM or MWCNTs alone (Fig 5A and 5B). Staining of lung sections with an Alcian blue and Periodic acid-Schiff (AB/PAS) combination stain demonstrated that mice sensitized with HDM allergen had notably increased goblet cell hyperplasia and mucin production, but that effect was not exacerbated by MWCNT exposure (S1 Fig). BALF collected from the lungs of mice was analyzed for secreted IL-1β after exposure to MWCNTs with or without HDM sensitization. IL-1β in the BALF was significantly increased at 1 day post-MWCNT exposure and remained elevated at 21 days compared to vehicle control, while sensitization of mice to HDM prior to MWCNT exposure almost completely inhibited IL-1β protein levels in BALF (Fig 5C). However, pro-IL-1β mRNA was increased in the lungs of mice at 1 day in all treatment groups compared to control and was most pronounced in mice exposed to HDM and MWCNTs (Fig 5D).

### Mice sensitized with HDM allergen have increased lung mRNA levels of IL-13 and IL-5, but not IL-4, and also have increased levels of serum IgE

**Fig 6:**
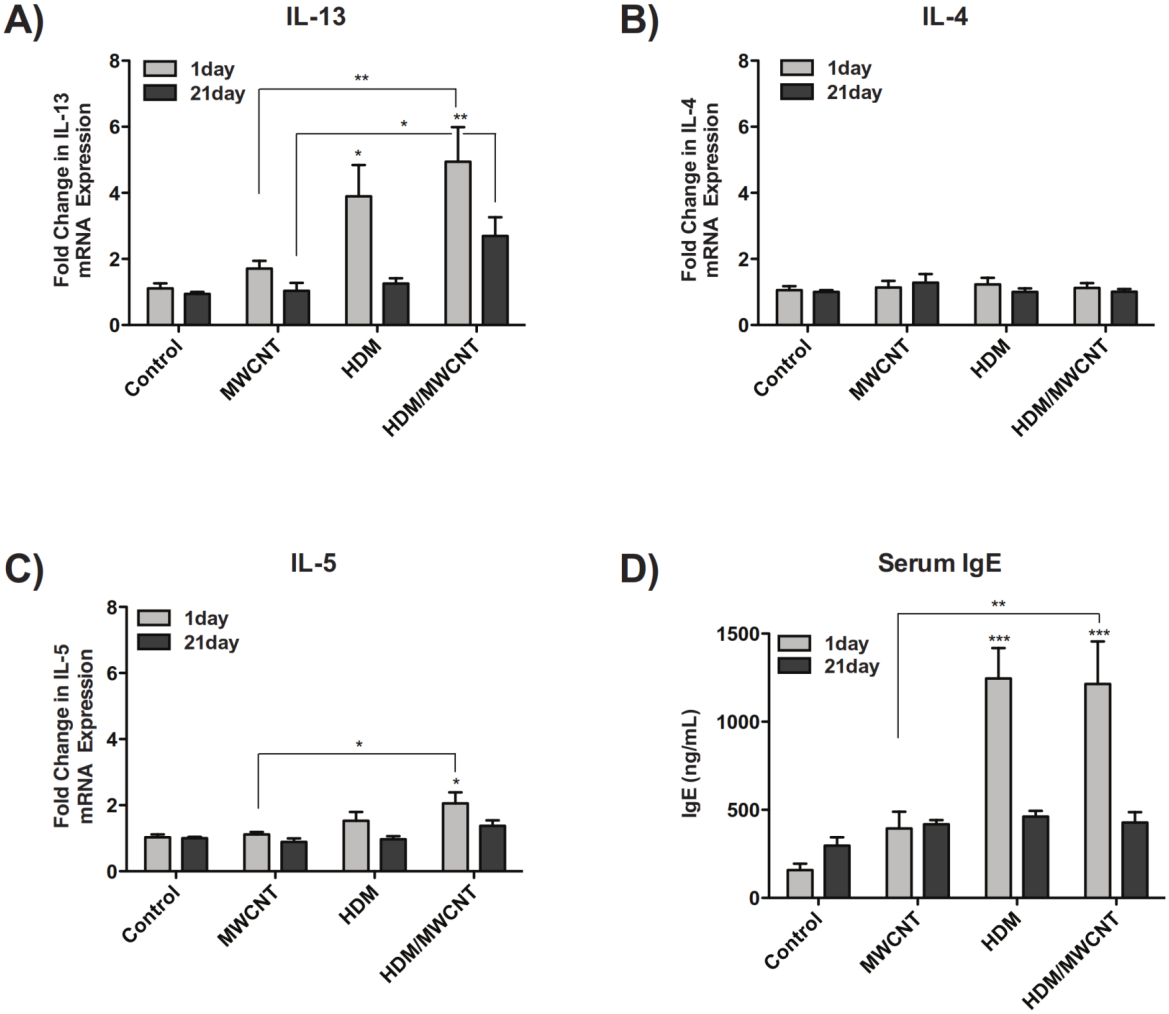
The Th2 response in mice following HDM sensitization and exposure to MWCNTs. (A) Lung mRNA levels of IL-13 at 1 day and 21 days in mice sensitized with HDM allergen both in the absence and presence of MWCNTs. (B) Lung mRNA expression of IL-4. (C) 1 day and 21 day expression of IL-5 mRNA in the lung. Statistical analysis performed using an unpaired student t-test. **P < 0.01, *P < 0.05. Values directly above bars indicate a significant difference compared to control animals. Values above connecting lines indicate a significant difference between MWCNT and HDM/MWCNT treatment. (D) Serum IgE levels at 1 day and 21 days. Statistical analysis performed using a one-way ANOVA with a *post-hoc* Tukey. ***P < 0.001 in treatment groups compared to control animals. **P < 0.01 in MWCNT-exposed animals compared to HDM/MWCNT treatment. N = 11–14 animals for all 1 day data. N = 8–13 animals for all 21 day data.

Sensitization of mice to HDM allergen significantly increased whole lung mRNA levels of IL-13 compared to control mice at 1 day post-MWCNT exposure (Fig 6A). Treatment of HDM-sensitized mice with MWCNTs up-regulated IL-13 mRNA expression even further compared to control mice and mice treated with only MWCNTs at 1 day and 21 day time points. IL-4 mRNA levels were not changed by HDM, MWCNT or the combination of HDM and MWCNT exposure at either 1 day and 21 days (Fig 6B). IL-5 mRNA levels were significantly increased at 1 day in mice sensitized to HDM and exposed to MWCNTs compared to control mice and mice exposed to MWCNTs alone (Fig 6C). Consistent with an allergic Th2 response, mice sensitized to HDM had increased serum IgE at 1 day compared to control, and exposure to MWCNTs did not change HDM-induced IgE levels (Fig 6D). Interestingly, MWCNTs alone caused a modest increase in serum IgE levels. No distinct increases in serum IgE were seen in any treatment groups at 21 days.

### HDM sensitization increases MWCNT-induced lung mRNA levels of the monocyte chemoattractant, CCL2, but does not alter mRNAs encoding neutrophil chemoattractants CXCL1 and CXCL2

**Fig 7:**
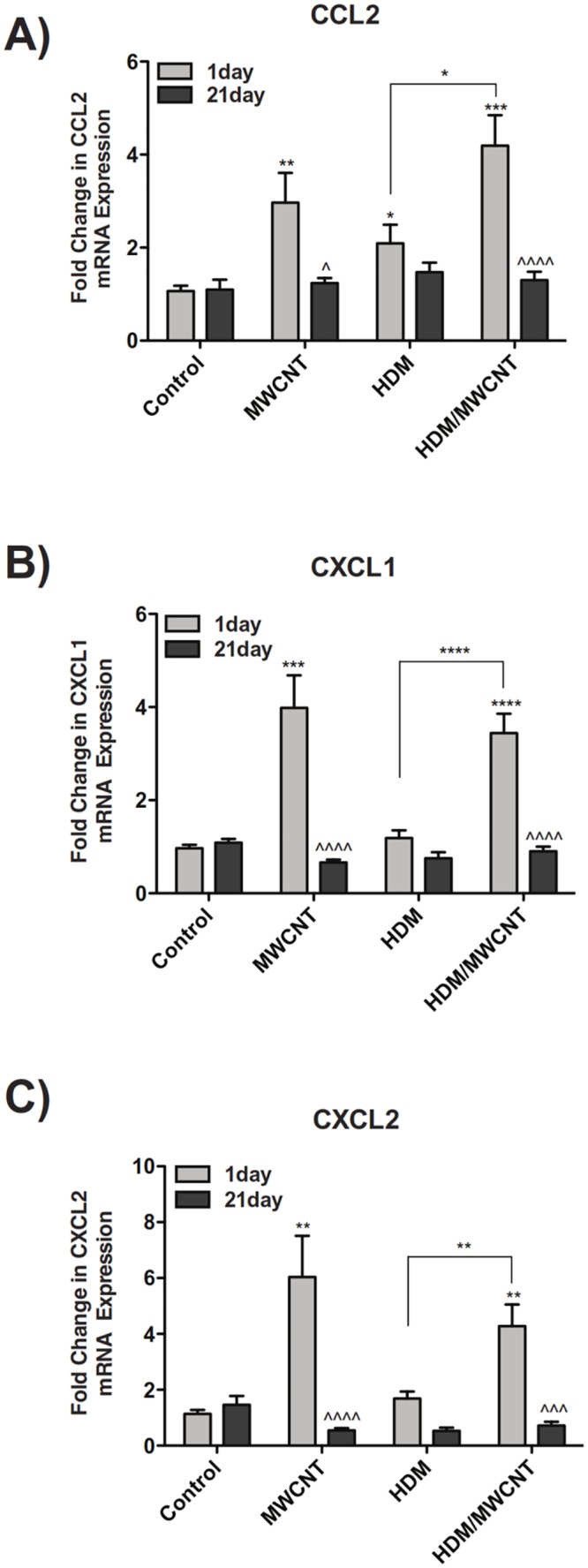
Lung mRNA levels of chemokines following HDM sensitization and exposure to MWCNTs. (A) Lung mRNA levels of CXCL1 at 1 day and 21 days in mice sensitized with HDM allergen with or without MWCNT exposure. (B) Lung mRNA expression of CXCL2. (C) 1 day and 21 day mRNA expression of CCL2 in the lung. Statistical analysis performed using an unpaired student t-test. *P < 0.05, **P < 0.01, ***P < 0.001, ****P < 0.0001 for comparison between treatment groups. ^^^P < 0.001. ^^^^P < 0.0001 for comparison between timepoints. N = 11–14 animals for all 1 day results. N = 8–13 animals for all 21 day results.

MWCNTs increased the expression of CCL2 mRNA, and the combination of HDM sensitization followed by MWCNT exposure further increased CCL2 mRNA (Fig 7A). MWCNTs significantly increased lung mRNA expression of CXCL1 (Fig 7B) and CXCL2 (Fig 7C) compared to control animals. While there was a trend for MWCNT-induced CXCL1 and CXCL2 mRNA levels to be suppressed by HDM pre-sensitization, these chemokine mRNA levels were not significantly different between MWCNT and HDM/MWCNT groups. HDM sensitization alone did not affect CXCL1 or CXCL2 mRNA levels.

### Pro-caspase-1 protein levels in the lungs of mice are induced by MWCNTs but suppressed by HDM sensitization at 1 day

**Fig 8:**
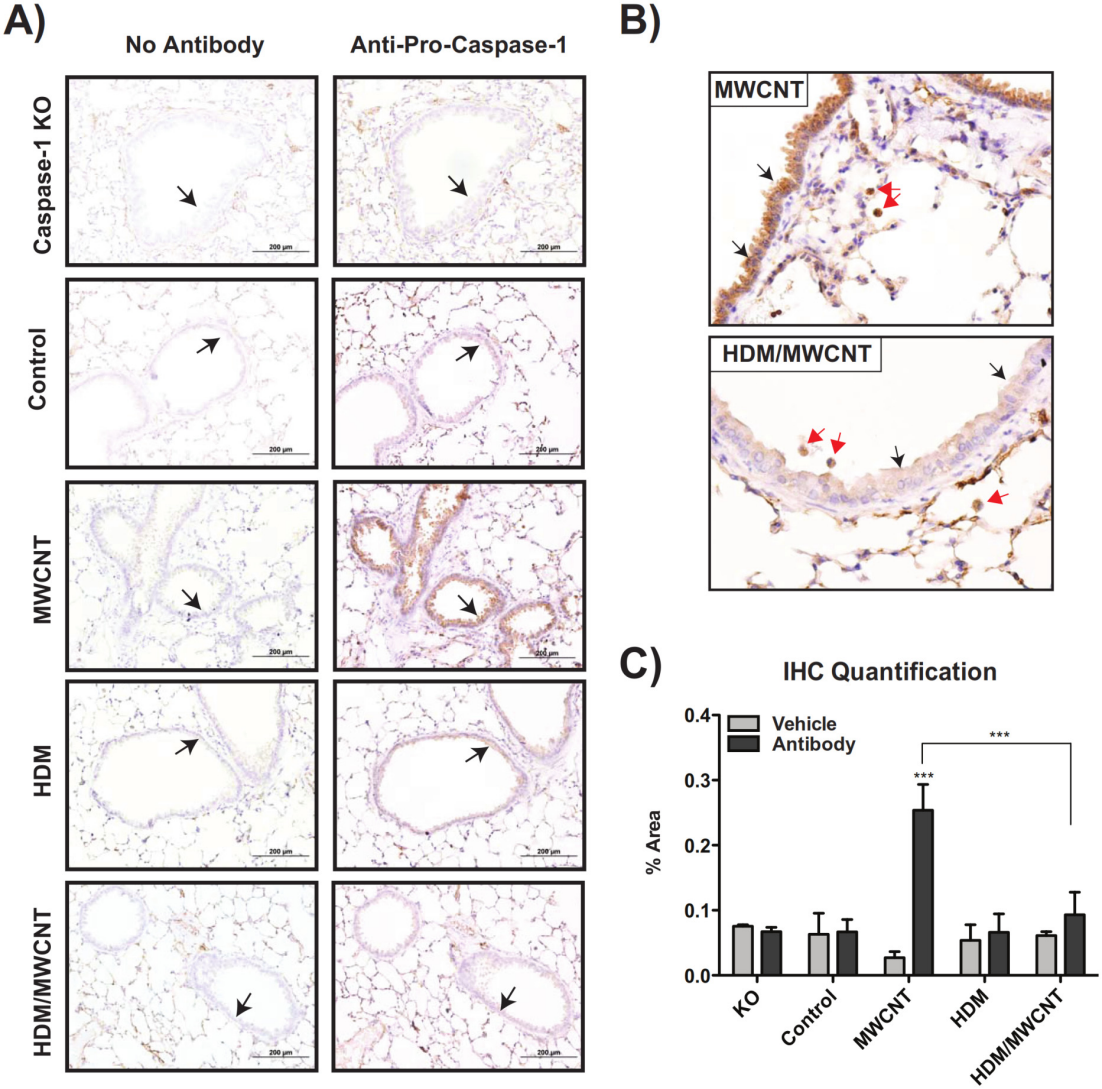
Pro-caspase-1 protein levels at 1 day in the lungs of mice are induced by MWCNTs but suppressed by HDM sensitization. Formalin-fixed, paraffin-embedded lung sections stained with an anti-pro-caspase-1 antibody or a vehicle control. Lung sections from caspase-1 knock-out (KO) mice were used as a negative control. (A) Pro-caspase-1 immuno-staining was observed in the airway epithelium at low magnification (20X). (B) Higher magnification (40X) images showing airway epithelium and alveolar macrophages staining. (C) Quantification of pro-caspase-1 staining in lung sections. Data is pooled from measurements of three individual airways per treatment group. Statistical analysis performed using a one-way ANOVA with a *post-hoc* Tukey. ***P < 0.001. Values directly above bars indicate a significant difference compared to control animals. Values above connecting lines indicate a significant difference between MWCNT and HDM/MWCNT treatments.

Pro-caspase-1 protein was visualized in the lungs of mice by immunohistochemical (IHC) staining. HDM sensitization did not increase levels of pro-caspase-1 compared to control mice that received vehicle alone, whereas oropharyngeal aspiration of MWCNTs caused pro-caspase-1 staining in airway epithelium (Fig 8A and 8B). Lung tissue from caspase-1 knock-out mice was used as a negative control for pro-caspase-1 immunohistochemistry. Mice that were sensitized to HDM and then exposed to MWCNTs had weaker immuno-staining for pro-caspase-1 in airway epithelium and alveolar macrophages as compared to mice that were exposed to MWCNTs alone (Fig 8A and 8B). Quantification of pro-capsase-1 IHC staining further supported the qualitative results observed, demonstrating a significant increase in pro-caspase-1 staining with MWCNTs alone, and that increase being suppressed with the combination of HDM and MWCNTs (Fig 8C).

### MWCNT exposure in mice following sensitization with HDM allergen amplifies airway fibrosis at 21 days

**Fig 9:**
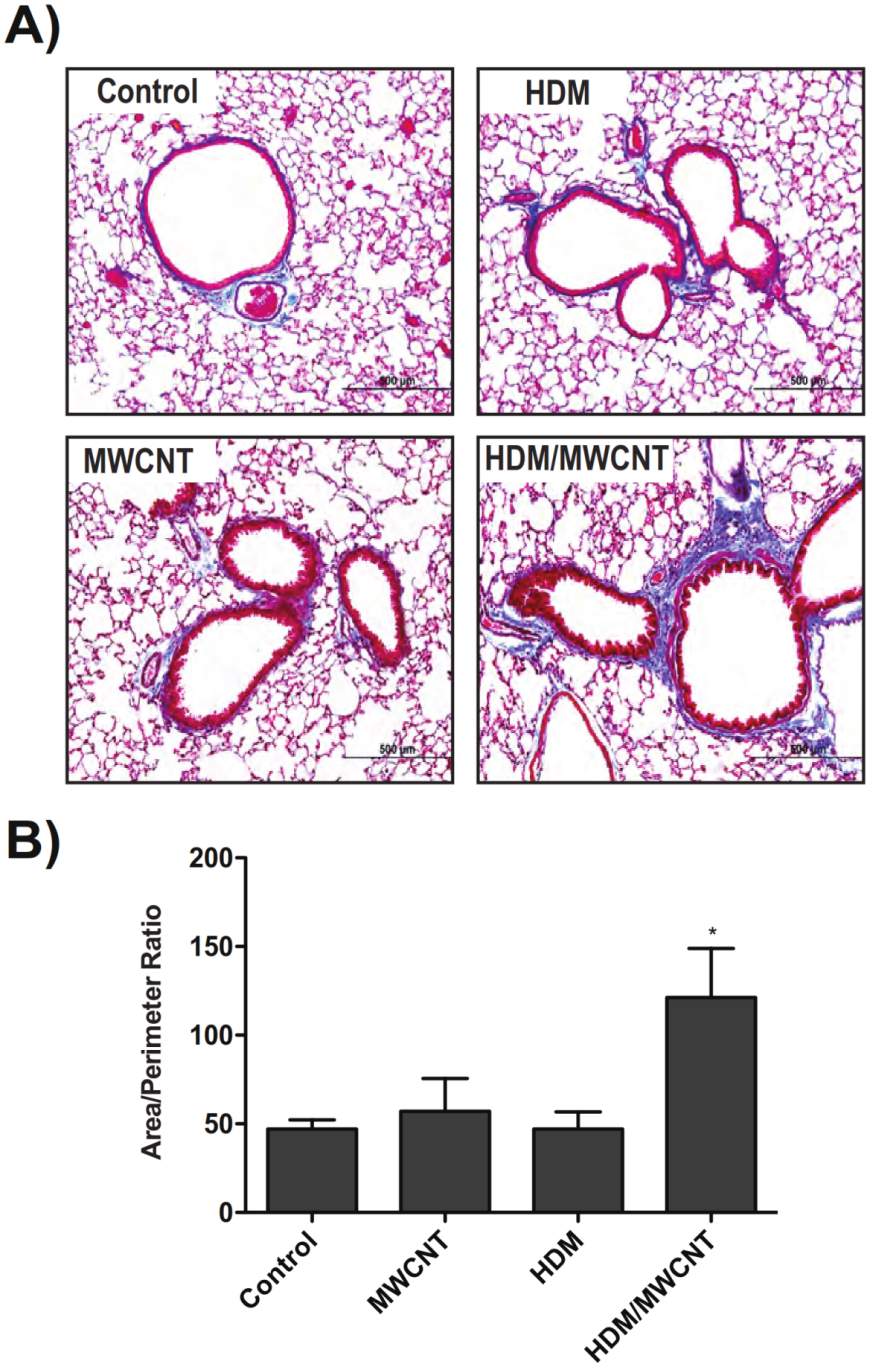
Airway fibrosis at 21 days post-exposure to MWCNTs is increased by pre-sensitization of mice with HDM allergen. (A) Representative lung sections stained with Masson’s trichrome stain in mice sensitized to HDM and then exposed to MWCNTs. Images taken at 10X magnification. (B) Semi-quantitative morphometric analysis of airway fibrosis. Data is pooled from 4–8 individual airway measurements. Statistical analysis performed using an unpaired student t-test. *P < 0.05 compared to control animals.

Masson’s trichrome staining of lung sections indicated increased collagen and airway fibrosis in mice treated with HDM and MWCNTs compared to MWCNTs or HDM alone (Fig 9A). Semi-quantitative morphometric analysis of airway area-perimeter ratio demonstrated a notable increase in airway fibrosis in mice exposed to MWCNTs after sensitization to HDM (Fig 9B). Taqman real-time quantitative RT-PCR showed a significant increase in lung mRNA levels of the pro-fibrogenic cytokines PDGF-A, PDGF-B, and TGF-β1 with MWCNTs alone or with combined HDM and MWCNT exposure (S2 Fig). However, the largest increases in these pro-fibrogenic cytokines was seen with combined HDM and MWCNT exposure.

## Discussion

**Fig 10:**
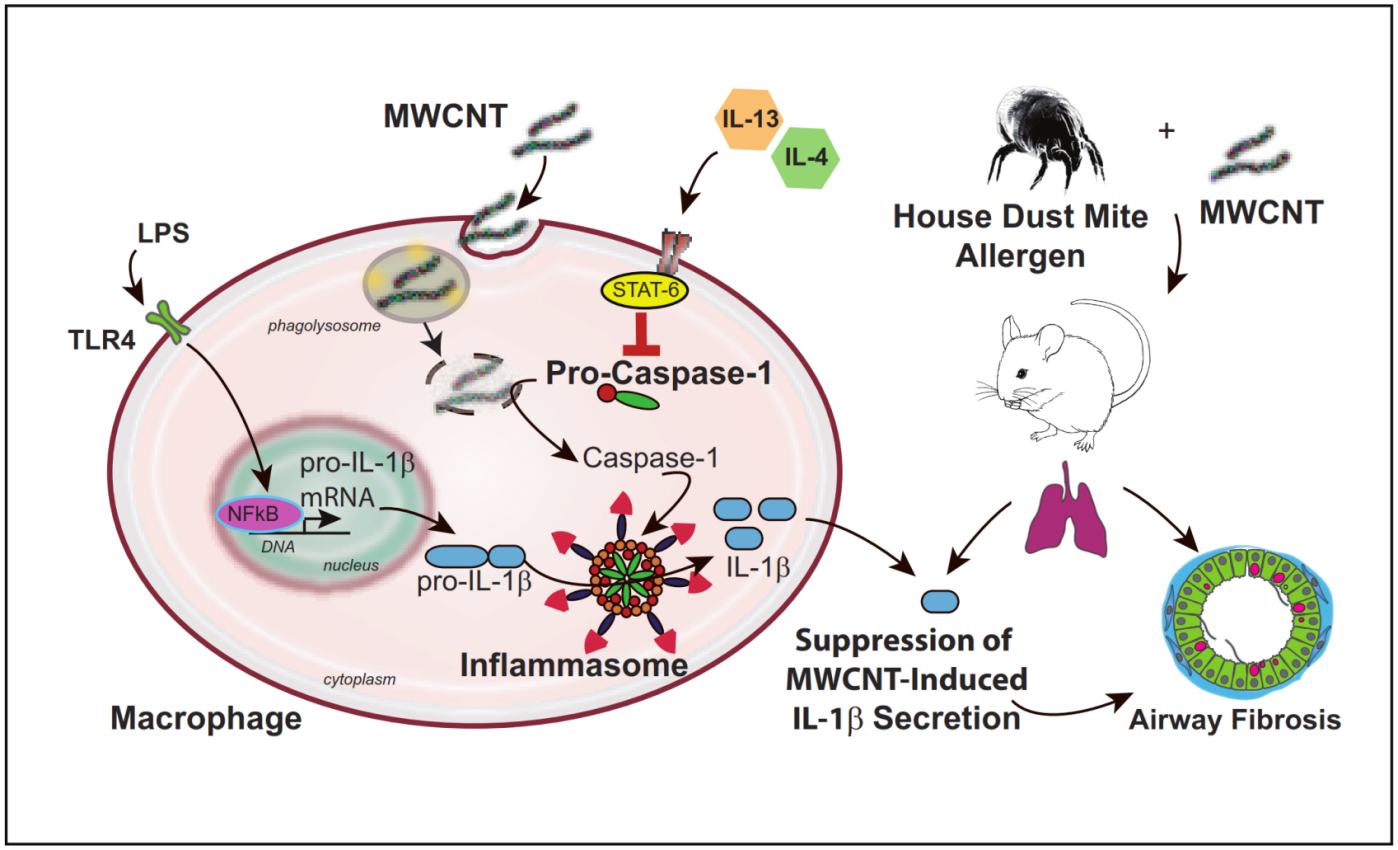
Schematic illustration of a hypothetical mechanism for suppression of MWCNT-induced inflammasome activation and IL-1β secretion during allergic airway inflammation and fibrosis. *In vitro* experiments using THP-1 monocytic cells differentiated to macrophages showed that the Th2 cytokines IL-4 and IL-13 suppress levels of intracellular pro-caspase-1 and reduce MWCNT-stimulated IL-1β secretion without affecting levels of pro-IL-1β. *In vivo* experiments with mice pre-sensitized to house dust mite allergen also showed suppression of MWCNT-induced IL-1β and pro-caspase-1 in the lungs, and revealed that MWCNT-induced airway fibrosis was enhanced by allergen challenge. Collectively, *in vitro* and *in vivo* experiments suggest that MWCNT-induced inflammasome suppressed in an allergic inflammatory microenvironment could play a role in increased airway fibrogenesis.

A number of studies have shown that MWCNTs activate the NLRP3 inflammasome in macrophages and epithelial cells to generate secretion of IL-1β, a key pro-inflammatory mediator in the lung [22,38,39]. We have previously reported that airway fibrosis induced by MWCNTs is exaggerated in the lungs of mice with pre-existing allergen-induced airway inflammation [5]. However, to our knowledge there are no reports that demonstrate how the allergic inflammatory microenvironment modulates MWCNT-induced inflammasome activation. Inflammasome activation and increased IL-1β have been implicated in the pathogenesis of pulmonary fibrosis [38,39]. However, we observed significantly more severe airway fibrosis in the lungs of mice exposed to MWCNTs after HDM allergen sensitization, even though IL-1β production was clearly suppressed in this experimental group. Our data support a mechanism of inflammasome suppression by an allergic microenvironment that is due at least in part to Th2 cytokine activation of STAT6 down-regulating intracellular levels of pro-caspase-1 (Fig 10). Our findings also suggest that suppression of inflammasome activation and IL-1β by an asthma microenvironment promotes the fibrogenic potential of MWCNTs.

The Th2 cytokines IL-4 and IL-13 bind to the IL-4/IL-13R to activate STAT-6, which plays a major role in mediating a variety of phenotypic endpoints in allergic airway inflammation, including eosinophilia, mucous cell metaplasia, and airway fibrosis [12,40]. We observed that IL-13 and IL-4 reduced levels of pro-capsase-1 mRNA and protein as demonstrated by qRT-PCR and Western blot analysis, respectively. IL-4 and IL-13 did not affect other inflammasome components in THP-1 cells, including ASC and NLRP3. Moreover, inhibitors of STAT6, specifically Leflunomide or JAK Inhibitor I (JAKI), blocked the suppression of active caspase-1 activity by IL-4 and IL-13. Moreover, Leflunomide and JAKI increased basal levels of caspase-1 activity in THP-1 cells, suggesting some constitutive activation of STAT6 in these cultured macrophages. These data are the first to our knowledge that demonstrate that Th2 cytokines interfere with NLRP3 inflammasome function by suppressing caspase-1 through a STAT6-dependent mechanism.

Importantly, our *in vitro* cell culture observations with THP-1 cells were supported by data obtained using a mouse model of HDM allergen-induced airway inflammation, where we showed that MWCNT-induced IL-1β production in the lungs of mice was significantly reduced by pre-sensitization with repeated HDM allergen exposure. Using immunohistochemistry, we showed that lung protein levels of pro-caspase-1 were reduced in the airway epithelium and macrophages in mice exposed to the combination of HDM allergen and MWCNTs, which indicated that suppression of pro-caspase-1 is a mechanism that contributes to inhibition of MWCNT-induced inflammasome activation *in vitro* and *in vivo* during allergic airway inflammation. These results are consistent with recent work that showed caspase-1-dependent IL-1β secretion was impaired in monocytes from patients with atopic dermatitis compared to healthy controls [28].

It is known that a combination of two signals is necessary to fully activate the NLRP3 inflammasome. This two-step process involves 1) a pathogen-associated molecular pattern (PAMP) binding to a pattern recognition receptor (PRR), and 2) phagocytosis of a foreign agent that disrupts the lysosomal membrane and releases cathepsin B to trigger assembly of the inflammasome components (NLRP3, ASC, caspase-1). A classic PAMP activator for signal 1 is bacterial LPS, which increases intracellular levels of pro-IL-1β [23,41]. In order to mimic this *in vitro*, we primed THP-1 cells with LPS immediately prior to MWCNT exposure. THP-1 cells are a human monocytic cell line that can be differentiated to a macrophage phenotype and are commonly used as a relevant cell model for *in vitro* responses to nanomaterials [32,42]. Western blotting showed that pro-IL-1β was highly induced by LPS. However, the Th2 cytokines IL-4 and IL-13 did not reduce levels of pro-IL-1β, indicating that the mechanism involved in suppression of MWCNT-induced IL-1β in THP-1 cell supernatants by IL-4 or IL-13 did not involve the first step of inflammasome activation involving a PAMP-induced increase in pro-IL-1β. Levels of pro-IL-1β mRNA were increased *in vivo* following exposure to MWCNTs, HDM, or the combination of HDM and MWCNTs at 1 day. It is currently unclear as to what acts as the priming signal *in vivo*, although preliminary research suggests that extracellular high-mobility group box 1 (HMGB1) may function *in vivo* similarly to LPS *in vitro* and aid in regulation of MWCNT-induced inflammasome activation [43].

The exact mechanism that mediates step two of the NLRP3 inflammasome activation process by particles and fibers is not well understood, but the main theory suggests that activation is dependent on recognition of lysosomal contents by the NLR protein following cell damage. Phagocytosis of fiber-like high-aspect ratio particles by macrophages leads to cellular damage and subsequent lysosomal rupture, during which lysosomal contents are released into the cytoplasm. Once in the cytoplasm, the NOD-like receptor (NLR) of the inflammasome, which is part of the family of PRRs, recognizes the lysosomal contents and activates pro-caspase-1 to caspase-1, which is then able to process pro-IL-1β and pro-IL-18 into mature, secretable forms [25]. The results of our *in vitro* work demonstrated that the Th2 cytokines were functioning in a manner that suppressed levels of pro-caspase-1. The action of these Th2 cytokines would suggest that an asthmatic phenotype, commonly associated with Th2 responses, would alter the activity of inflammasomes following MWCNT exposure. Levels of caspase-1 can be measured in terms of pro-caspase-1 mRNA and protein by qPCR and Western blot, respectively, or in terms of cleaved, mature caspase-1, via an enzymatic activity assay. We performed all of these techniques, and achieved cohesive results for levels of both pro-caspase-1 and mature, active caspase-1.

A recent translational study has shown that expression of inflammasome genes is decreased in samples of sputum inflammatory cells from patients with allergic rhinitis or asthma [31]. Using a panel of markers of innate immunity that included inflammasome components, Brickey and colleagues showed that patients with asthma or allergic rhinitis had significantly suppressed innate immune responses. Data from their experiments showed decreased pro-caspase-1, pro-IL-1β, and ASC mRNA levels in both asthmatic patients and patients with allergic rhinitis [31]. Our *in vitro* data using THP-1 cells showed a similar trend for pro-caspase-1 mRNA levels decreased by Th2 cytokine treatment, but we did not observe suppression of pro-IL-1β, NLRP3, or ASC mRNA levels by Th2 cytokines in THP-1 cells. Nevertheless, our research supports the translational human study by Brickey and colleagues by demonstrating that an allergic Th2 microenvironment suppresses inflammasome activation.

Our *in vitro* experiments with THP-1 were predictive of the *in vivo* HDM model of allergic airway inflammation, in that we observed that a Th2 microenvironment suppressed pro-caspase-1 and MWCNT-induced secretion of IL-1β in both model systems without inhibiting the expression of pro-IL-1β. However, the HDM sensitization mouse model allowed us to better understand the pathological consequences of inflammasome suppression on acute inflammation and chronic airway remodeling. Interestingly, despite suppressed levels of secreted IL-1β in the BALF of mice exposed to HDM prior to MWCNT exposure, we observed an enhanced lung inflammatory cell infiltration in this treatment group. To further correlate our *in vitro* work to *in vivo* observations, we examined mRNA levels of Th2 cytokines in the lungs of exposed mice to determine if increased levels of IL-13, IL-4, or IL-5 could be responsible for the suppression of MWCNT-induced IL-1β in HDM-sensitized mice. IL-13 and IL-5 mRNA levels were increased in mice sensitized to HDM alone and in mice sensitized to HDM and exposed to MWCNTs at both 1 day and 21 days. However, IL-4 mRNA expression was not elevated in any treatment group. This could be due to a different temporal expression pattern of IL-4 compared to IL-13 or perhaps IL-13 plays a more significant role than IL-4 *in vivo*. Due to the nature of our HDM exposure protocol, it is possible that levels of Th2 cytokines in the BALF are increased during the two-week HDM sensitization window and levels of IL-4 could have returned to baseline after suppressing pro-caspase-1. Alternatively, since Th2 cells are the major source of IL-13 and IL-4, it is possible that most of the detectable IL-13 and IL-4 was compartmentalized in lymphatics and interstitium and not in the alveolar space that is lavaged during necropsy.

We observed increased airway fibrosis in the lungs of mice 21 days after exposure to MWCNTs, but only when animals were pre-exposed to HDM. Exposure to MWCNTs alone or HDM alone did not significantly increase airway fibrosis. Focal interstitial fibrotic lesions in the lung parenchyma were observed in either MWCNT or HDM/MWCNT treatment groups. Levels of PDGF-A, PDGF-B, and TGF-β1 lung mRNA were increased in mice exposed to MWCNTs both with and without prior HDM sensitization compared to control animals, although there was not a significant difference between the two treatments. PDGF and TGF-β1 have been implicated as playing major roles in fibrogenesis [44]. However, because PDGF-A and-B or TGF-β1 mRNA levels were similarly increased in MWCNT and HDM/MWCNT groups, other cytokines may be more important to the airway fibrogenic response observed after HDM and MWCNT exposure. Lung tissue levels of CCL2 mRNA, a pro-fibrogenic chemokine, were significantly induced at 1 day in the HDM/MWCNT group compared to MWCNTs alone. We have previously shown that CCL2 is important in mediating fibrosis in the lungs of mice exposed to nickel nanoparticles, although CCL2 also serves to reduce mucous cell metaplasia [16]. Other possible explanations could account for increased fibrosis in the HDM/MWCNT group, such as differences in collagen degradation or differences in the cytokine receptor expression in airway mesenchymal cells.

Our findings suggest that MWCNT-induced inflammasome activation and subsequent IL-1β production in the lungs of mice *in vivo* does not promote airway fibrosis in an allergic inflammatory setting, since significant airway fibrosis was observed only in mice that received HDM sensitization followed by MWCNT exposure, and these animals had suppressed levels of IL-1β in BALF. However, the role of IL-1β in lung inflammation and fibrosis is complex and some studies using MWCNTs, either *in vivo* or *in vitro*, have implicated IL-1β as a pro-fibrogenic cytokine. For example, Wang *et al* showed that more dispersed MWCNTs caused greater interstitial fibrosis *in vivo* in the lungs of mice and this corresponded to increased levels of a variety of cytokines, including IL-1β [38]. Hussain *et al* showed that exposure of cultured human airway epithelial cells *in vitro* to MWCNTs induced inflammasome activation and a subsequent increase in pro-fibrogenic markers (TIMP-1, Tenascin-C, Procollagen 1, and Osteopontin) in cultured human lung fibroblasts *in vitro*, and that these pro-fibrogenic marker responses were reduced by IL-1β neutralizing antibodies [39]. We recently reported that MWCNTs coated with a thin film of Al_2_O_3_ by atomic layer deposition caused less fibrosis in the lungs of mice compared to uncoated MWCNTs, and this corresponded to lower levels of IL-1β in BALF [42]. In this same study, Al_2_O_3_-coated MWCNTs stimulated higher levels of IL-1β secretion in THP-1 cells compared to uncoated MWCNTs, indicating that IL-1β production in cultured cells *in vitro* does not always predict IL-1β production *in vivo* in the lungs. Whether IL-1β is pro-fibrogenic or anti-fibrogenic could depend on temporal or spatial expression in tissues, or might depend on other factors produced in the inflammatory microenvironment.

While the total number of inflammatory cells in BALF was increased by the combination of HDM and MWCNTs compared to MWCNTs alone, the relative percentage of neutrophils in BALF was reduced by ~50% in animals exposed to HDM and MWCNTs relative to MWCNTs alone. This loss of neutrophil influx early in the inflammatory response could play a role in the development and exacerbation of fibrosis in the mice sensitized to HDM and exposed to MWCNTs. Levels of CXCL1 and CXCL2, both neutrophil chemoattractants, were increased at 1 day with MWCNT treatment, and that increase was suppressed with the combination of HDM and MWCNTs. These data suggest that IL-1β may be more important to the resolution of inflammation and that suppression of innate inflammation promotes a fibrogenic response in the lungs following MWCNT exposure. Other laboratories have demonstrated that MWCNTs delivered to the lungs of mice or rats, in the absence of any allergen sensitization, cause interstitial fibrotic lesions [45,46]. However, we have also previously reported that inhaled MWCNTs exacerbate airway fibrosis in mice pre-sensitized with ovalbumin [5]. Based on this data, we anticipated that MWCNTs alone would not induce a significant fibrotic response, but that the combination of HDM and MWCNTs would exacerbate airway fibrosis.

Research into the role of caspase-1 in allergic asthma has produced contradictory results. Using immunohistochemical staining, we discovered that levels of MWCNT-induced pro-caspase-1 are suppressed in mice previously sensitized to HDM allergen. These data supported our hypothesis that the Th2 microenvironment suppresses IL-1β secretion and the innate inflammatory response through inhibition of pro-caspase-1. The antibody used for IHC staining recognizes the 45kDa pro-caspase-1 protein, but also weakly binds the cleaved, 10kDa piece of mature caspase-1. Therefore it is possible that some of the staining observed in the lung sections may be mature caspase-1, but we believe the majority of staining is from immature pro-caspase-1. Other studies on allergic airway inflammation have been performed using *Casp1* ^*-/-*^ mice, albeit with differing results. Eisenbarth *et al*, discovered that ovalbumin (OVA) sensitization of *Casp1* ^*-/-*^ mice caused reduced airway inflammatory responses as compared to wild-type mice, whereas Willart *et al* found that *Casp1* ^*-/-*^ mice sensitized to HDM had levels of airway inflammation that more closely resembled wild type animals [47,48]. Bauer *et al* showed that *Casp1* ^*-/-*^ mice had reduced airway hyperresponsiveness (AHR) to methacholine challenge, but also had increased neutrophilic infiltration and airway inflammation, suggesting that caspase-1 may play opposing roles in allergic airway disease through 1) protection against airway inflammation, and 2) enhancement of allergen-induced AHR [49]. Our results showed that pro-caspase-1 is suppressed by HDM-induced allergic inflammation, which reduced inflammasome activation and subsequent IL-1β production. We also showed that relative numbers of neutrophils were decreased in the lungs of mice sensitized with HDM then exposed to MWCNTs compared to mice exposed to MWCNTs without HDM sensitization, even though total numbers of inflammatory cells were increased by combined HDM and MWCNT exposure.

## Conclusions

In summary, we demonstrated that allergic airway inflammation decreased inflammasome activity following MWCNT exposure in mice *in vivo* and that Th2 cytokines (IL-4 and IL-13) that characterize allergic airway inflammation suppressed the inflammasome in THP-1 cells *in vitro*. The mechanism involves Th2 cytokine suppression of pro-caspase-1, which is a key component of the inflammasome that converts pro-IL-1β to mature IL-1β. Our data suggests that asthma and other allergic airway diseases have the potential to suppress innate immune function via alteration of inflammasome action following MWCNT exposure. Therefore, individuals with asthma represent a susceptible population at risk for exposure to carbon nanotubes. Our data also support the idea that inflammasome suppression could impede the resolution of tissue remodeling to result in the development of pulmonary fibrosis. Further research needs to be done in order to fully understand the mechanisms through which Th2 cytokines suppress the innate immune response to MWCNTs.

## Supporting Information

S1 Fig Sensitization with HDM increases mucin production and goblet cell hyperplasia in the lungs of mice at 1 day, and MWCNTs do not exacerbate this effect. (A) Representative Alcian blue and Periodic acid-Schiff (AB/PAS)-stained lung sections from mice sensitized with HDM and exposed to MWCNTs at 1 day. Arrows in MWCNT panel indicate MWCNTs in the airway and alveolar space. Arrows in HDM and HDM/MWCNT panels indicate goblet cell hyperplasia. All images taken at 10X. (B) Semi-quantitative analysis of mucin production in the lungs of mice at 1 day post-MWCNT exposure. Statistical analysis performed using a one-way ANOVA with a *post-hoc* Tukey. *P < 0.05. Values directly above bars indicate significant difference from control mice. Values above connecting lines indicate significant difference compared to MWCNT treatment. (C) Semi-quantitative analysis of mucin production in the lungs of mice at 21 days post-MWCNT exposure. Statistical analysis performed using a one-way ANOVA with a *post-hoc* Tukey. *P < 0.05 for treatment groups compared to control animals. N = 11–14 animals for 1 day results. N = 8–13 animals for 21 day results.
(TIF)

S2 Fig MWCNTs enhance the lung fibrotic response at 21 days in mice sensitized with HDM allergen. 1 day and 21 day lung mRNA levels of pro-fibrotic growth factors in mice sensitized with HDM allergen in the absence or presence of MWCNTs. (A) Lung PDGF-A mRNA expression. (B) 1 day and 21 day PDGF-B levels. C) TGF-β1 mRNA expression at 1 and 21 days. Statistical analysis performed using an unpaired student t-test. ***P < 0.001, **P < 0.01, *P < 0.05 for treatment groups compared to control and for comparison in-between treatments. N = 11–14 animals for all 1 day data. N = 8–13 animals for all 21 day data.
(TIF)
