# Supplementary figures and images for: An Allergic Lung Microenvironment Suppresses Carbon Nanotube-Induced Inflammasome Activation via STAT6-Dependent Inhibition of Caspase-1
Source: PLoS One. 2015 Jun 19;10(6):e0128888. doi: 10.1371/journal.pone.0128888 (PMC4474696; doi:10.1371/journal.pone.0128888)

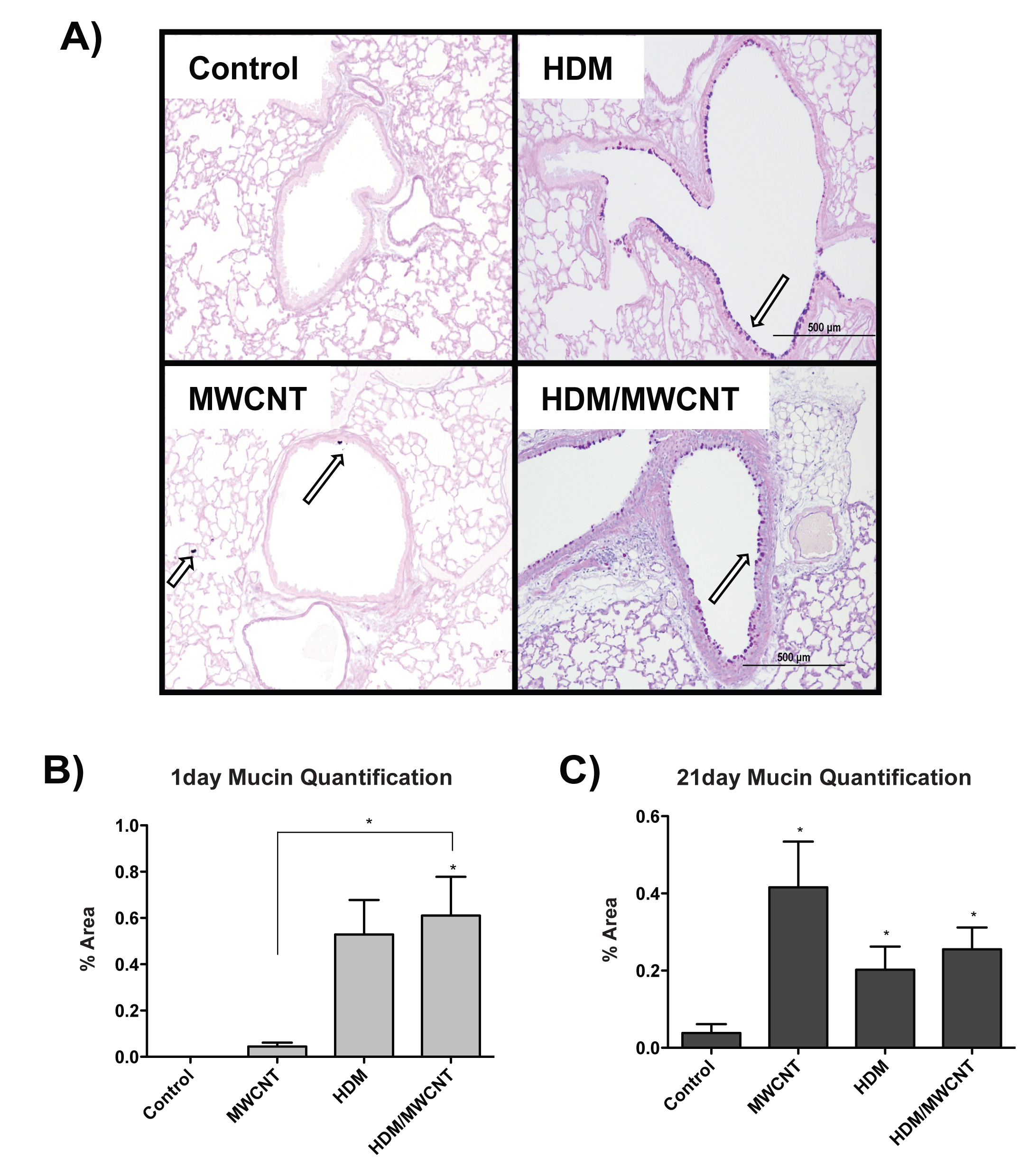

Supplement: S1 Fig — (A) Representative Alcian blue and Periodic acid-Schiff (AB/PAS)-stained lung sections from mice sensitized with HDM and exposed to MWCNTs at 1 day. Arrows in MWCNT panel indicate MWCNTs in the airway and alveolar space. Arrows in HDM and HDM/MWCNT panels indicate goblet cell hyperplasia. All images taken at 10X. (B) Semi-quantitative analysis of mucin production in the lungs of mice at 1 day post-MWCNT exposure. Statistical analysis performed using a one-way ANOVA with a post-hoc Tukey. *P < 0.05. Values directly above bars indicate significant difference from control mice. Values above connecting lines indicate significant difference compared to MWCNT treatment. (C) Semi-quantitative analysis of mucin production in the lungs of mice at 21 days post-MWCNT exposure. Statistical analysis performed using a one-way ANOVA with a post-hoc Tukey. *P < 0.05 for treatment groups compared to control animals. N = 11–14 animals for 1 day results. N = 8–13 animals for 21 day results. (TIF) [file pone.0128888.s001.tif]

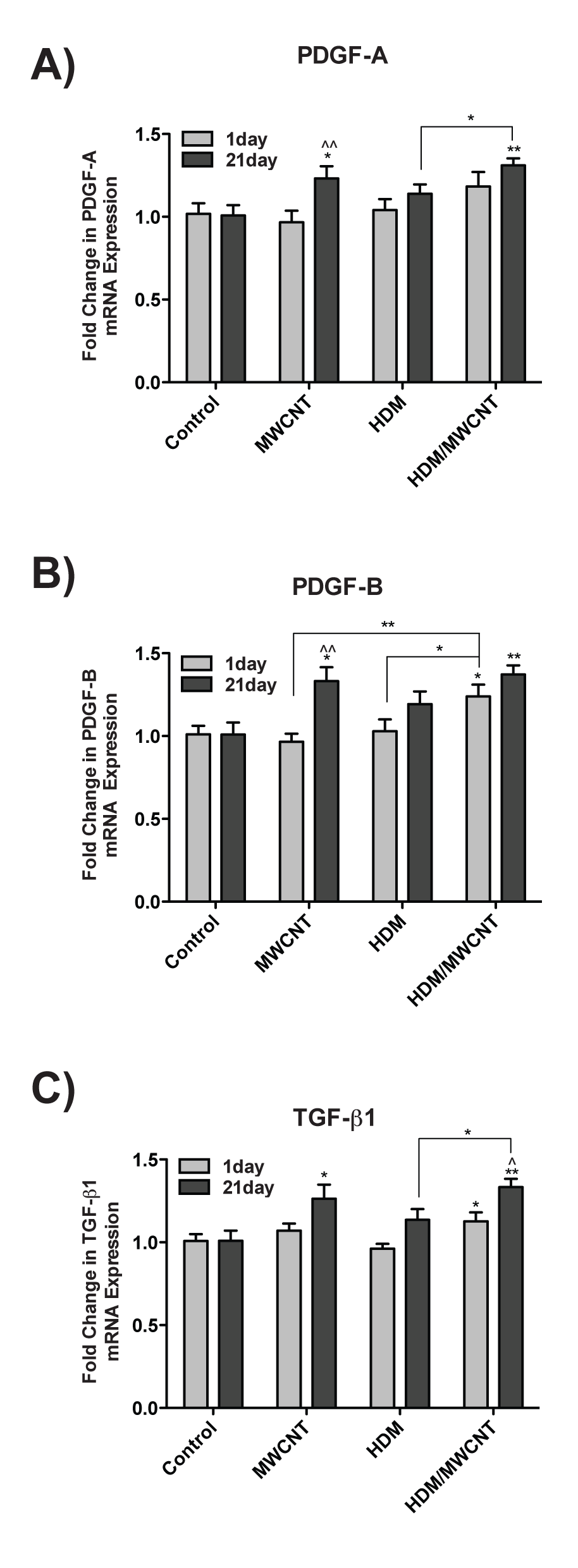

Supplement: S2 Fig — 1 day and 21 day lung mRNA levels of pro-fibrotic growth factors in mice sensitized with HDM allergen in the absence or presence of MWCNTs. (A) Lung PDGF-A mRNA expression. (B) 1 day and 21 day PDGF-B levels. C) TGF-β1 mRNA expression at 1 and 21 days. Statistical analysis performed using an unpaired student t-test. ***P < 0.001, **P < 0.01, *P < 0.05 for treatment groups compared to control and for comparison in-between treatments. N = 11–14 animals for all 1 day data. N = 8–13 animals for all 21 day data. (TIF) [file pone.0128888.s002.tif]
